# Multimorbidity and patient experience with general practice: A national cross-sectional survey in Norway

**DOI:** 10.1186/s12875-024-02495-1

**Published:** 2024-07-10

**Authors:** Rebecka Maria Norman, Elma Jelin, Oyvind Bjertnaes

**Affiliations:** Norwegian Institute of Public Health, PO Box 222, Skøyen, Oslo, NO-0213 Norway

**Keywords:** Primary health care, Physicians, GP, General practice, Patient experiences, Survey, Psychometrics

## Abstract

**Background:**

Patient experience is an important indicator of the quality of healthcare. Patients with multimorbidity often face adverse health outcomes and increased healthcare utilisation. General practitioners play a crucial role in managing these patients. The main aim of our study was to perform an in-depth assessment of differences in patient-reported experience with general practice between patients living with chronic conditions and multimorbidity, and those with no chronic conditions.

**Methods:**

We performed secondary analyses of a national survey of patient experience with general practice in 2021 (response rate 41.9%, *n* = 7,912). We described the characteristics of all survey respondents with no, one, two, and three or more self-reported chronic conditions. We assessed patient experience using four scales from the Norwegian patient experience with GP questionnaire (PEQ-GP). These scales were used as dependent variables in bivariate and multivariate analyses and for testing the measurement model, including confirmatory factor analysis and a multigroup CFA to assess measurement invariance. Sentiment and content analysis of free-text comments was also performed.

**Results:**

Patients with chronic conditions consistently reported lower scores on the GP and GP practice experience scales, compared to those without chronic conditions. This pattern persisted even after adjustment for patient background variables. The strongest associations were found for the scale of “Enablement”, followed by the scales of “GP” and “Practice”. The subscale “Accessibility” did not correlate statistically significantly with any number of chronic conditions. The analysis of free-text comments echoed the quantitative results. Patients with multimorbidity stressed the importance of time spent on consultations, meeting the same GP, follow-up and relationship more often than patients with no chronic conditions. Our study also confirmed measurement invariance across patients with no chronic conditions and patients with multimorbidity, indicating that the observed differences in patient experience were a result of true differences, rather than artifacts of measurement bias.

**Conclusions:**

The findings highlight the need for the healthcare system to provide customised support for patients with chronic conditions and multimorbidity. Addressing the specific needs of patients with multimorbidity is a critical step towards enhancing patient experience and the quality of care in general practice.

## Introduction

People living with chronic conditions are a large and growing group of patients and users of primary healthcare in Norway [1], and internationally [2]. The severity level varies with the combination of chronic conditions and with age. The co-existence of two or more chronic conditions is often described as multimorbidity [3, 4]. Patients with multimorbidity are a heterogeneous group with varying diagnoses and a number of coexisting conditions; however, multimorbidity will have an impact on both the individual, such as increased risk of death [5], reduced quality of life and functioning [6, 7], and at a healthcare provider and health system level, such as a greater risk of hospitalisation and readmission [8], and an increase in primary care consultations and total healthcare costs [9, 10].

General practitioners (GPs) play a crucial role in managing patients with chronic conditions and multimorbidity [9, 10]. The GP scheme in Norway entitles all inhabitants the right to choose a designated GP. This scheme is grounded on the idea that having a designated GP ensures access and continuity, which are important indicators of quality [11]. The GP scheme in Norway is facing challenges, marked by an increasing number of patients without a designated GP. Patients without a designated GP are at risk of delayed diagnoses, insufficient follow-up care, and overall poorer health outcomes. This situation is particularly worrisome for vulnerable individuals and those living with multiple health conditions [12]. Similar challenges related to GP practice have been described in other countries such as the UK [13]. These challenges can have a major impact on the quality of healthcare for this group of patients.

Patient-reported experience, measured through surveys is important when evaluating aspects of healthcare quality [14]. It is of interest to explore whether the poorer outcomes found in previous studies [5, 7–9] of patients with multimorbidity are reflected in self-reported experience of care in this group. Some evidence supports this hypothesis; for example, a study of patient’s experience with GP practice in England found that patients with multimorbidity more frequently reported poorer experiences with care [15], and one American study found lower scores of doctor-patient communication among people with several chronic conditions [16]. However, the research base is limited, and we know little about how patients with chronic conditions and multimorbidity experience GP practice, compared to the experience of patients with no chronic conditions in Norway. As the situation of increasing demands on GP practice is similar internationally, the results will be applicable to healthcare systems similar to Norway’s. A better understanding of differences in experience can be used to adapt healthcare to the needs of the patients and thereby improve care.

The main objectives of this study were to perform an in-depth assessment of differences in patient-reported experience with general practice between patients living with chronic conditions and multimorbidity (two or more chronic conditions), compared to patients living with no chronic condition. This includes to (i) quantitatively explore possible differences; (ii) analyse free-text comments to provide complementary information on possible differences between the groups; and (iii) examine whether potential differences in patient experience were due to true differences in scores (measurement invariance), or whether the constructs were not psychometrically equivalent across patients with no chronic conditons and those with two or more chronic conditions/multimorbidity.

## Methods

We used data from a national representative survey of patient experience with GPs and GP practices that was carried out in Norway in 2021–2022. The national survey was commissioned by the Norwegian Ministry of Health and Care Services and conducted by the Norwegian Institute of Public Health (NIPH).

### Setting

In Norway, as in several other countries, the GP is the gatekeeper to more specialised healthcare. All residents in Norway are entitled to a regular GP as a part of the regular GP scheme, with around 99% of the population being part of it [17]. GP practices are generally organised in small units with 2–5 GPs, most of whom are self-employed [18].

### Sampling and data collection

GP practices were first stratified according to the size of the municipality and the number of GPs in the practice, and secondly, we randomly selected 2,000 GPs within these practices. We lacked information about 52 of these GPs. In the last step, ten patients were randomly selected from the lists of selected GPs (*N* = 19,480). The inclusion criteria were patients aged 16 years and older with a minimum of one contact with the GP in the last 12 months. Eligible patients registered in a national digital portal received a digital invitation to the survey with an electronic response option, while others received a postal invitation letter with an electronic response option. Non-responders were sent two reminders by post, with the option to respond on paper or electronically [19].

### Patient experience measures

The Norwegian patient experience with GP questionnaire (PEQ-GP) consists of five subscales: assessment of GP (8 items), cooperation (2 items), patient enablement (3 items), accessibility (2 items) and practice (3 items). The cooperation subscale is mostly relevant for patients with chronic conditions and has items missing above 20% [19, 20], and hence, this scale was excluded from this article. The questions underlying each scale are presented in Table 1. The response format ranges from “not at all” to “to a very great extent”, with the additional response of “not applicable” [21]. These five response categories were scored as 0, 25, 50, 75 and 100, respectively.

**Table 1:** Demographic and health characteristics of respondents according to no, one, two, and three or more self-reported chronic conditions (*n* = 7,701)

	No chronic condition	One chronic condition	Multimorbidity: two chronic conditions	Multimorbidity: three or more chronic conditions
	*n* = 2,269	*n* = 2,661	*n* = 1,580	*n* = 1,191
	*n* (%)	*n* (%)	*n* (%)	*n* (%)
**Sex**		n.s	n.s	n.s
Male	953 (42.0)	1,165 (43.8)	689 (43.6)	511 (42.9)
Female	1,316 (58.0)	1,496 (56.2)	891 (56.4)	679 (57.0)
**Age in groups (years)**		***	***	***
16–49	950 (41.9)	762 (28.6)	363 (23.0)	209 (17.6)
50–66	691 (30.5)	921 (34.6)	567 (35.9)	457 (38.4)
67–79	482 (21.2)	777 (29.2)	512 (32.4)	349 (29.3)
80+	146 (6.4)	201 (7.6)	138 (8.7)	175 (14.7)
**Education**		***	***	***
Primary school	272 (12.1)	331 (12.6)	226 (14.4)	247 (20.9)
High school	711 (31.5)	930 (35.3)	589 (37.6)	457 (38.7)
University, ≤ 4 years	667 (29.6)	785 (29.8)	462 (29.5)	307 (26.0)
University, ≥ 4 years	604 (26.8)	585 (22.2)	291 (18.6)	169 (14.3)
**Country of birth**		***	***	**
Norway	1,895 (83.7)	2,363 (89.0)	1,417 (89.9)	1,027 (86.5)
Western countries including: Europe within the EU/North America/Oceania	210 (9.3)	174 (6.6)	94 (6.0)	71 (6.0)
Non-western countries including: Eastern Europe outside the EU/Asia/Africa/South America	159 (7.0)	119 (4.5)	66 (4.2)	89 (7.5)
**Self-reported physical health**		***	***	***
Very poor/rather poor	13 (0.6)	145 (5.5)	165 (10.5)	286 (24.1)
Both poor and good	166 (7.3)	664 (25.0)	604 (38.3)	578 (48.6)
Rather good/very good	2,089 (92.1)	1,848 (69.6)	808 (51.2)	325 (27.3)
**Self-reported mental health**		***	***	***
Very poor/rather poor	27 (1.2)	94 (3.5)	98 (6.2)	120 (10.1)
Both poor and good	227 (10.0)	446 (16.8)	344 (21.8)	326 (27.5)
Rather good/very good	2,013 (88.8)	2,116 (79.7)	1,135 (72.0)	738 (62.3)

Difference between group and respondents with no chronic conditions: *p < 0.05, **p < 0.01, ***p < 0.001, n.s: not statistically significant

### Variables

The PEQ-GP includes questions on participants’ characteristics, including the item “Do you have a long-lasting condition? (defined as lasting six months or longer, or new conditions expected to be long-lasting)”, with the response options: “yes, one”, “yes, two”, “yes three or more” and “no”. This item was used for categorisation of chronic conditions.

The PEQ-GP also includes the items: country of birth (in categories), education, self-perceived physical and mental health, whether they usually see their own GP, time since last contact with GP, and waiting time for regular and urgent appointments. Age, sex, number of consultations in the last 24 months, and years on the GP list, were collected from registry data.

### Statistical analyses

We described the characteristics of all survey respondents with no, one, two, and three or more self-reported chronic conditions, and evaluated differences between the respondents with chronic conditions and respondents reporting no chronic conditions using chi-square and t-tests. For bivariate analysis we used analysis of variance (ANOVA) and t-tests.

The potential need for a multilevel approach was assessed by estimating the intraclass correlation coefficient (ICC) and the design effect statistics on the four scales. We used an estimated design effect above two as an indication of the need for multilevel (nested) models in the analyses [22].

Multiple linear regression was carried out, with patient experience with GP as dependent variables (four scales), and chronic conditions or not (1, 2 and 3/more vs. 0) as an independent variable, adjusting for patient sex, age, country of birth and education. For all tests conducted, a *p* < 0.05 was assigned as the level of statistical significance.

We used confirmatory factor analysis (CFA) to evaluate a generic four-factor subscale structure. The full information maximum likelihood estimation method (FIML) were used to account for missing data, with estimation that used all cases, including cases missing one or several items [23]. Model fit was evaluated using the root mean square error of approximation (RMSEA), the standardized root mean squared residual (SRMR), and the comparative fit index (CFI). RMSEA values of 0.05 or less and SRMR of 0.08 or less are considered a close fit, and CFA of 0.95 or greater is considered acceptable [24].

We evaluated measurement invariance with multigroup CFA because it is found to be common to have non-equivalent psychometric constructs (measurement non-invariance) across disease groups and chronic conditions in generic instruments [25]. Measurement invariance assesses the psychometric equivalence of a construct across groups, which is essential when testing mean differences.

Measurement invariance was evaluated by comparing patients reporting no chronic conditions with patients reporting two or more chronic conditions. We also evaluated measurement invariance between patients with no chronic condition and one chronic condition, no chronic condition and two chronic conditions, and between no chronic condition and three or more chronic conditions.

We followed the approach described by Putnick and Bornstein [26], fitting models with a progressively more stringent set of equity constraints, using four hierarchical steps: (1) *configural invariance*: the factor structure is the same across groups; (2) *metric invariance*: factor loadings are similar across groups; (3) *scalar invariance*: equivalence of item intercepts and thresholds across groups; and (4) *strict invariance*: equivalence of items’ residuals. There is no consensus concerning the best-fit indices or cut-off values [26], and we used a common criterion proposed by Chen [27] who suggested a criterion of a -0.01 change in CFI paired with changes in RMSEA of 0.015, and SRMR of 0.030, and the invariance at that hierarchical level should be rejected. If the model is invariant, results can be compared directly across the groups. As a robustness test, we also tested the model fit using robust (Huber-White) standard errors and a scaled test statistic (MLM), and listwise deletion of missing values.

Data analyses were performed with IBM SPSS Statistics for Windows, Version 25.0 software (Armonk, NY: IBM Corp.), while confirmatory factor analysis and multigroup CFA were conducted using R version 3.6.3 (packages: lavaan).

### Analyses of free-text comments

The questionnaire included a free-text field where respondents were invited to write more about their experience with their GP and GP practice. To complement the quantitative analyses and contextualise responses to structured questions, two researchers (RMN and EJ) independently coded 100 free-text comments from patients with multimorbidity and 100 free-text comments from patients without any self-reported chronic conditions. The starting point for deciding on the number of comments was a previous study from our research group, utilizing a sample size of 50 comments per group [28]. To secure a robust material we doubled the number to 100 comments per group. During data analysis, we reached data saturation well within 100 comments, which showed that additional data would no longer provide new insights. The free-text comments were analysed for sentiment as positive, negative, mixed or of neutral character. Sentiment analysis of free-text comments aligns with the newest methods for analysing complementary open-ended patient experience data [29].

Comments that did not address the GP services or healthcare quality were excluded from the content analyses. Content was coded in an iterative manner based on major themes and subthemes in the free-text comments. When new themes emerged, the coding structure was revised, and the previous comments were re-read to determine congruence. Some themes were not divided into a subtheme. Any coding ambiguities were resolved through joint discussion. Lastly, the comments were sorted according to multimorbidity or not, and sentiments and themes identified in the comments were compared between the groups.

## Results

After removing people with insufficient addresses, people who made reservations against participation, and deceased persons, the main sample consisted of 18,861 patients. The total number of responses was 7,912, with a total response rate of 41.9%. 7,701 respondents gave an answer to the question of whether they had a chronic condition, and of these 2,269 responded with no chronic condition.

Respondent characteristics according to the number of chronic conditions are presented in Table 1. Compared to respondents without chronic conditions, patients with one, two, or three or more chronic conditions were more likely to be older (age 50–80+), with lower levels of education, and lower levels of self-perceived physical and mental health. For country of birth, there was a lower proportion of non-Norwegian patients with one or two chronic conditions. There were no differences in sex (Table 1).

**Table 2:** Use of the GP and the GP practice according to no, one, two, and three or more self-reported chronic conditions (*n* = 7,701)

	No chronic condition	One chronic condition	Multimorbidity: two chronic conditions	Multimorbidity: three or more chronic conditions
	*n* = 2,269	*n* = 2,661	*n* = 1,580	*n* = 1,191
	*n* (%)	*n* (%)	*n* (%)	*n* (%)
**Usually see own GP**		n.s	n.s	n.s
Yes	1,933 (87.2)	2,348 (88.8)	1,368 (87.3)	1,055 (89.5)
No	285 (12.8)	295 (11.2)	199 (12.7)	124 (10.5)
**Time since last contact with GP**		***	***	***
Less than 1 month ago	689 (30.5)	1,122 (42.3)	754 (47.9)	678 (57.0)
1–3 months ago	662 (29.3)	864 (32.5)	495 (31.4)	341 (28.7)
4–6 months ago	463 (20.5)	390 (14.7)	209 (13.3)	112 (9.4)
More than 7 months ago	448 (19.8)	279 (10.5)	117 (7.4)	59 (5.0)
**Waiting time for ordinary appointment**		**	***	***
0–3 days	646 (31.3)	721(29.0)	370 (25.0)	296 (27.0)
4–7 days	636 (30.8)	708 (28.5)	453 (30.6)	311 (28.4)
8–14 days	501 (24.3)	626 (25.2)	408 (27.5)	291 (26.5)
More than 2 weeks	282 (13.7)	428 (17.2)	251 (16.9)	199 (18.1)
**Waiting time for urgent appointment**		n.s	n.s	n.s
Same or next day	1,161 (63.4)	1,365 (62.4)	799 (61.7)	368 (62.9)
After 2 days	236 (12.9)	292 (13.3)	173 (13.4)	110 (10.8)
After more than 2 days	433 (23.7)	531 (24.3)	322 (24.9)	266 (26.2)
**Number of consultations in the last 24 months**		***	***	***
	2,269 (mean = 4.5)	2,661 (mean = 6.7)	1,580 (mean = 8.2)	1,190 (mean = 10.1)
**Years on GP list**		n.s	n.s	n.s
	2,269 (mean = 8.0)	2,661 (mean = 8.1)	1,580 (mean = 8.3)	1,191 (mean = 7.7)

Difference between group and respondents with no chronic conditions: *p < 0.05, **p < 0.01, ***p < 0.001, n.s: not statistically significant

Use of the GP and the GP practice according to the number of chronic conditions are presented in Table 2. Compared to respondents without chronic conditions, patients with one, two, or three or more chronic conditions were more likely to have had a longer waiting time for an ordinary appointment, last consultation more previously, and more consultations with the GP. There were no differences in waiting time for urgent appointments, years on the GP list, and whether they usually saw their own GP (Table 2).

**Table 3:** Mean scores (SD) for patient experience with the GP and the GP practice scales and items according to no, one, two, and three or more self-reported chronic conditions

	No chronic condition Mean (SD)	One chronic condition Mean (SD)	Multimorbidity: two chronic conditions Mean (SD)	Multimorbidity: three or more chronic conditions
**GP**	**78.7 (16.9)**	76.8 (17.7) *	76.1 (18.3) ***	74.6 (19.5) ***
GP takes you seriously	82.7 (19.5)	81.8 (20.2)	81.8 (21.1)	79.8 (22.9) ***
GP spends enough time with you	73.8 (23.2)	71.8 (24.0)	71.7 (24.0)	70.1 (25.6) ***
GP talks to you in a way you understand	84.3 (17.7)	83.6 (18.1)	83.3 (18.8)	81.5 (20.3) ***
GP is professionally competent	81.2 (18.6)	80.1(19.5)	79.8 (19.7)	78.8 (20.6) **
GP shows interest in your situation	78.5 (21.0)	77.3 (21.9)	77.2 (22.5)	75.4 (23.9) ***
GP includes you as much as you would like in decisions concerning you	78.5 (20.2)	77.5 (20.3)	77.1 (21.8)	75.6 (22.4) ***
GP provides sufficient information about health problems and treatment	77.3 (20.5)	74.9 (21.9) **	73.0 (23.1) ***	72.2 (23.9) ***
GP provides sufficient information about use/side effects of medication	69.2 (26.4)	65.8 (26.0) ***	64.2 (26.0) ***	62.7 (27.8) ***
**Practice**	**78.9 (18.1)**	**78.9 (18.0)**	**79.2 (17.4)**	**79.2 (18.2)**
GP practice well organised	76.6 (20.2)	76.6 (20.8)	76.3 (20.9)	75.9 (21.1)
Other employees are helpful and competent	79.1 (20.3)	79.3 (19.5)	79.8 (19.3)	79.6 (20.4)
Treated with courtesy and respect in reception	80.9 (20.6)	80.8 (20.6)	81.6 (19.3)	82.4 (19.4)
**Accessibility**	**63.9 (27.4)**	**63.6 (27.4)**	**62.9 (26.9)**	**61.3 (29.2)**
Waiting time for your last urgent appointment acceptable	69.6 (30.8)	70.1 (30.7)	69.6 (30.2)	66.7 (33.3)
Waiting time for appointments that are not urgent acceptable	59.3 (29.7)	58.0 (29.8)	57.3 (29.5)	56.5 (30.4)
**Enablement**	**71.8 (21.4)**	67.9 (22.2) ***	66.9 (22.3) ***	64.0 (24.0) ***
Contact with GP make you better able to understand your health problems	73.9 (22.5)	70.2 (23.5) ***	69.5 (23.9) ***	67.1 (25.5) ***
Contact with GP make you better able to cope with your health problems	72.1 (22.9)	67.1 (24.1) ***	65.5 (24.7) ***	63.1 (26.4) ***
Contact with GP better helps you to stay healthy	69.7 (24.6)	66.3 (24.8) ***	65.6 (24.2) ***	61.6 (26.1) ***

Analysis of variance (ANOVA). Difference between respondents with no chronic condition and other groups: *p < 0.05, **p < 0.01, ***p < 0.001

### Bivariate analysis

Mean scores for the four scales and the underlying items are shown in Table 3. Patients with one, two, and three or more chronic conditions reported a statistically significantly, and decreasingly lower score on the “GP” scale and the “Enablement” scale, compared to those without chronic conditions. Those with three or more chronic conditions reported consistently lower scores for all eight items on the “GP” scale, compared to those without chronic conditions. Patients with one, two, and three or more chronic conditions reported a significant and decreasingly lower score for the items: “GP provides sufficient information about health problems and treatment” and “GP provides sufficient information about use and side effects of medication.” Patients with one, two, and three or more chronic conditions reported a significant and decreasingly lower score for all items within the “Enablement” scale, compared to those without chronic conditions. There were no differences for other items.

**Table 4:** Associations between chronic conditions and patient experience with the GP and GP practice scale scores, adjusted for patient-level characteristics

	GP		Practice		Accessibility		Enablement	
	B	95% CI	B	95% CI	B	95% CI	B	95% CI
One chronic condition (ref: no chronic condition)	-2.135***	**-3.15, -1.12**	-1.11*	**-2.12, -0.10**	-0.68	-2.26, 0.91	-4.38***	**-5.81, -2.95**
Two chronic conditions (ref: no chronic condition)	-2.14***	**-3.93, -1.59**	-1.35*	**-2.51, -0.18**	-1.27	-3.10, 0.56	-5.50***	**-7.10, -3.90**
Three or more chronic conditions (ref: no chronic condition)	-3.90***	**-5.19, -2.60**	-1.64*	**-2.92, -0.36**	-1.98	-4.01, 0.05	-8.55***	**-10.29, -6.82**
Adjustment variables								
Male (ref: female)	0.36	-0.45, 1.18	0.80	-0.1, 1.61	0.30	-0.98, 1.58	3.18***	**2.08, 4.28**
Age	0.04**	**-0.02, 0.06**	0.20***	**0.18, 0.23**	0.03	-0.01, 0.06	0.10***	**0.07, 0.13**
Western countries (ref: Norway)	-0.72	-2.28, 0.85	0.18	-1.39, 1.75	-3.58**	**-6.02, -1,13**	-0.49	-2.60, 1.61
Non-western countries (ref: Norway)	-4.56***	**-6.34, -2.78**	-2.90**	**-4.68, -1.11**	-10.95***	**-13.73, -8,18**	1.62	-0.69, 3.93
Education	1.28***	**0.86, 1.70**	-0.30	-0.72, 0.12	2.38***	**1.72, 3.03**	0.41	-0.16, 0.97

From multiple linear regressions: *p < 0.05, **p < 0.01, ***p < 0.001. Statistically significant estimates in bold

### Multivariate analysis

The ICC ranged from 0.09 (Enablement scale) to 0.14 (Practice scale). None of the scales had a design effect above two, which supported the appropriateness of not accounting for the nested (multilevel) design in the analysis. Assumptions for conducting a linear regression were fulfilled.

After adjustment for patient sex, age, country of birth, and education in the multiple linear regression models the negative association between patient experience scale scores and number of chronic conditions remained, showing the same, and an even stronger, trend as in the bivariate analysis (Table 4). The strongest correlations were found for the “Enablement” scale, followed by “GP” and “Practice”. The “Accessibility” subscale did not correlate statistically significantly with any number of chronic conditions. The regression coefficients showed that with an increasing number of chronic conditions, the patient experience scores decreased.

There were some differences between the impact of different background variables used for adjustment to explain the patient experience. For all scales except “Enablement”, patients with a non-western birth country had more negative experiences compared to patients born in Norway. On the “Accessibility” scale, patients born in western countries also had statistically significantly lower scores, compared to patients born in Norway. With increasing age, patients had significantly better experience for all scales except “Accessibility”, which did not correlate statistically significantly with age. Higher education was significantly associated with a higher score on the “GP” and “Accessibility” scales. Males had a significantly better score for “Enablement” compared to females.

### Confirmatory factor analysis and measurement invariance

The four-factor structure had an acceptable fit for data in the entire sample (RMSEA = 0.054, SRMR = 0.040, CFI = 0.972). Results from a single group CFA supported an acceptable fit for both patients with no chronic conditions and patients with multimorbidity. Results from multigroup testing between these groups support that the model is measurement invariant, and thus, means can be compared between these groups. Results are shown in Table 5. Results from multigroup testing between the other groups (no chronic condition and one chronic condition, no chronic condition and two chronic conditions, and between no chronic condition and three or more chronic conditions) showed that all the models were measurement invariant with acceptable fit statistics (results not shown). For robustness testing, we conducted the tests using a robust estimation method (MLM), and the results showed a good fit of data, with almost similar fit statistics as when using FIML (results not shown).

**Table 5:** Results from confirmatory factor analysis and measurement invariance testing

	Chi-squared statistics	Degrees of freedom	Comparative Fit Index (CFI)	Root Mean Square Error of Approximation (RMSEA)	Standardized Root Mean Squared Residual (SRMR)
**Overall sample**	2387,778	98	0.972	0.054	0.040
**Single group**					
No chronic condition	723.444	98	0.971	0.053	0.038
Multimorbidity	932.724	98	0.973	0.055	0.041
**Multiple group**					
Configural	1,656.168	196	0.972	0.054	0.039
Metric	1,700.66	208	0.972	0.053	0.042
Scalar	1,813.509	220	0.970	0.054	0.043
Strict	1,889.401	236	0.968	0.053	0.043

Estimation method: Full information maximum likelihood

### Free-text comments

The free-text comment field was used by *n* = 938 (34%) patients with multimorbidity and *n* = 568 (25%) patients with no self-reported chronic conditions. For analysis, *N* = 100 comments were randomly selected in each group. The free-text comments were first analysed for sentiment. In the group with multimorbidity, there were 40 positive comments, 32 negative, 19 mixed, and 9 of neutral character. In the group with no chronic conditions, there were 44 positive comments, 27 negative, 18 mixed, and 11 of neutral character.

Second, we coded for thematic content. The following themes, in order, were most addressed in the group with multimorbidity: accessibility, communication/relational aspects followed by continuity, professionalism, and organisational aspects, while some comments expressed general satisfaction with the GP. The most commonly positive subthemes were related to an expression of general satisfaction with the GP, followed by being taken seriously by the GP, professionalism and meeting the same GP. The negative subthemes mostly concerned the time spent on the consultation, not meeting the same GP, and use of substitute GPs, followed by organisational aspects. One participant wrote: “My GP now has a substitute for him, so I don’t know how it will turn out. It’s fine to go via e-consultation to make an appointment, but hopeless to call.” Several patients expressed concern that their GP was about to quit or retire, and they did not have a say in the situation. One participant wrote: “Have had the same GP for 38 years and he has followed me and my three children until now. Dreading his retirement soon. It will be difficult to open up about everything to a completely new person.”

Patients with multimorbidity addressed most often, in order, the subthemes time spent on consultations, and meeting the same GP more often than patients with no chronic conditions. One participant wrote: *“I have had the same GP since the scheme started, and it has been exactly how I think and feel it should be. Very confident that I will get the help and advice that is best for me. Very pleased!”*. Another participant expressed the opposite about not having the same GP: *“One problem is that my GP always has someone who substitutes for him, and I don’t meet the person I have had as a GP for 25 years.”* Other themes were follow-up and relationship which patients with multimorbidity addressed more often than patients with no chronic conditions.

In the group with no chronic conditions, the most commonly positive subthemes were the same as for the group of patients with multimorbidity. The negative subthemes mostly concerned the use of substitute GPs, communication, long waiting times, digital accessibility, and organisational aspects. Patients without multimorbidity addressed the subthemes of waiting time and use of substitute GPs more often than patients with multimorbidity. The comments related to the use of substitute GPs were both positive and negative from the group with no chronic conditions, while this subtheme was only negatively addressed by the group of patients with multimorbidity.

## Discussion

In this study, we investigated associations between the self-reported number of chronic conditions and patient experience with GP and GP practice in Norway. The general picture showed that even after adjusting for patient sex, age, country of birth, and education, on most patient experience scales patients with chronic conditions scored the GP and GP practice statistically significantly more poorly compared to patients with no chronic condition. The strongest associations were found for the “Enablement” scale, followed by “GP” and “Practice”. The results from the analysis of the free-text comments showed the same overall results and add to the understanding of the poorer experience of patients with chronic conditions and multimorbidity. Multigroup CFA showed that these lower scores among patients with chronic conditions were due to true differences in mean scores.

Paddison, Saunders [15] found similar results in a large UK study, where patients’ experience of primary care declined with an increasing number of chronic conditions. There are several possible explanations. People with several chronic conditions and multimorbidity have more complex needs than those with single or no chronic conditions. It has been previously argued that these complex needs may not be covered well enough by a system of healthcare delivery that is designed for patients with a single condition [30]. The system is designed for the patient to limit the consultation time of approximately 15 min to one medical issue/query and to make a new appointment for any further conditions. The extent of this practice varies among GP practices in Norway [31]. This system is commonly used internationally, and it is argued that these organisational practices can result in a fragmented experience of care and an increased burden of treatment, especially among patients with chronic conditions and multimorbidity [32]. The importance of a healthcare system that considers patients with chronic conditions is demonstrated by previous studies. A recent Norwegian study found, for example, higher mortality associated with lower continuity for patients with COPD, diabetes mellitus and heart failure [33].

The results from the analysis of use of the GP and GP practice showed that, compared to respondents without chronic conditions, patients with one, two, or three or more chronic conditions were more likely to have had a longer waiting time for an ordinary appointment. This is somewhat surprising, given that a patient’s health status can change rapidly for those with chronic conditions and multimorbidity. However, it might be the case that patients with chronic conditions prefer continuity and to wait to be able to see their own GP, compared to patients with no chronic condition. We found no differences in waiting time for urgent appointments. A study of patient preferences found that patients with chronic conditions were willing to accept a longer waiting time to see the doctor of choice [34] and there is evidence that older patients are more likely to have a preference for a certain GP [35]. These results add to the understanding that patients with chronic conditions, when not urgent, prefer to wait for their own GP. This is also in line with that we found no differences between the groups related to whether the waiting time was acceptable. Our results from the free-text comments also showed the same tendency, as patients with multimorbidity addressed the time spent on consultations, meeting the same GP, follow-up and relationship more often than patients with no chronic conditions. The latter more often addressed waiting time for an appointment. We found no differences between the groups related to whether patients saw their own GP, but the relative impact on this matter may vary.

We found that both the “GP” and the “Enablement” scale, which consists of items on communication and care coordination, had the strongest association with the number of chronic conditions. Previous studies have found that patients with multimorbidity report better experience of care when they are knowledgeable and involved in the decision-making, when their care is well-coordinated, and when communication is good [36]. Multimorbidity may be associated with more medication, tests, and referrals that the GP, in turn, needs to discuss with the patient. Discussion of multiple and complex topics within a consultation may encourage the GP to employ communication strategies that leave patients feeling that their providers have not listened carefully, explained clearly, involved the patient in decision-making, demonstrated respect, or spent enough time with the patient [16].

On most scales, another birth country than Norway showed more negative experience, with the most negative scores found among patients born in non-western countries, with a decline in patient experience score as the number of chronic conditions increases. This is in line with previous studies that have reported differences in patient experience and general satisfaction with the GP, according to immigrant background [37, 38]. However, our results show that when this group has chronic conditions the experience declines even further.

Higher education was associated with a higher score on the “GP” and “Accessibility” patient experience scales. Education can be seen as a proxy for socioeconomic status. Socioeconomic deprivation is particularly associated with multimorbidity [39]. A previous study has found that higher socioeconomic status (measured as income) is associated with better experience and satisfaction with the GP and GP practice [38].

We did not adjust for health-related quality of life as, for example, Paddison, Saunders [15] did. We argue that such an adjustment is problematic, as adjustment variables should lie outside the control of the health services. Having a chronic condition suggests that the patient has lower self-perceived physical or mental health, compared to no chronic condition. Previous evidence has found an inverse relationship between the number of chronic conditions and health-related quality of life [7]. It is not surprising that the healthcare services will have an impact on patients’ health. For example, it is found that effective physician-patient communication can have an impact on health outcomes, including emotional health, and functional and physiological measures [40]. Adding to this, results from a meta-analysis show that the odds of patient adherence to medication were found to be 2.16 times higher if a physician had good communication skills [41]. Hence, we do not find health-related quality of life relevant to use as an adjustment variable in this study. Our results also showed that having a chronic condition entails more contact with the GP, and patients’ use of the GP is also something which the GP can have an impact on, for example more follow-ups if necessary for the condition and the patient.

### Implications

Future research using larger samples should consider possible variations and further explore patient experience relating to various combinations of factors. Additionally, to further investigate whether there are specific chronic conditions, or combinations of conditions, that are associated with patient experience concerning the GP. Moreover, continuity of care should be included and further explored in relation to chronic conditions and multimorbidity. This knowledge could be used to design interventions to improve the patient’s experience of the GP.

Although there may be some differences between countries in organising GP and primary health care services, our findings suggest that it is important for healthcare providers to adapt their practices to meet the unique needs of patients with chronic conditions. Even though a number of aspects related to the use of the GP and the GP practice had poorer scores for those with chronic conditions compared those without chronic conditions, the largest differences were related to enablement and information. Prioritising strategies and initiatives to improve empowerment, information, and self-management for persons with chronic conditions seems highly warranted both in education, practice, and policy. Furthermore, previous research shows that GPs need better multimorbidity management guidance and policies and practice models that secure finance trustful therapeutic partnerships [42]. Our results showed that patients with chronic conditions who were born in non-western countries had even more negative experience then patients born in Norway, with a decline in patient experience scores as the number of chronic conditions increased. Clinicians and policymakers might need to consider cultural competence training [43] and similar initiatives to improve experiences for minority chronic groups, while maintaining a balance of providing the same quality of care to all patients.

### Strengths and limitations

A strength of the study is the use of data from a large national survey using a questionnaire developed according to established procedures, with an inclusion of free-text comments from patients with and without multimorbidity. This approach provides useful information on possible differences between the groups and adds to knowledge about areas for quality improvements and policy. Furthermore, evaluation of the measurement model revealed that the model showed good fit statistics and measurement invariance, meaning that the scores can be compared between groups.

The overall response rate was 42%, meaning that some patient groups may be more representative than others. However, the response rate alone may not be the most critical factor determining possible non-response bias [44]. Another limitation is that the number and types of long-term conditions were self-reported by patients responding to the survey and were not cross-checked against patients’ clinical records. Furthermore, we approached long-term conditions as a number of conditions. However, this is a generic concept which disregards the great diversity among different types and the level of severity of the chronic condition and specific combinations.

## Conclusions

Across most patient experience scales, patients with chronic conditions consistently rated their GP and GP practice lower than those without chronic conditions. This was evident even when adjustments were made for patient sex, age, country of birth, and education. Analysis of free-text comments echoed the quantitative results. Patients with multimorbidity stressed the importance of time spent on consultations, meeting the same GP, follow-up and relationship more often than patients with no chronic conditions.

## Data Availability

The datasets generated during and/or analysed during the current study are available from the corresponding author on reasonable request.

## Abbreviations

CFA: Confirmatory factor analysis
CFI: Comparative fit index
FIML: Full information maximum likelihood
GP: General Practitioner
ICC: Intraclass correlation coefficient
PEQ-GP: The Norwegian patient experiences with GP questionnaire
RMSEA: Root mean square error of approximation
SRMR: Standardized root mean squared residual
